# Supplementary material for: Electrospun Fibers and Sorbents as a Possible Basis for Effective Composite Wound Dressings
Source: Micromachines (Basel). 2020 Apr 22;11(4):441. doi: 10.3390/mi11040441 (PMC7231366; doi:10.3390/mi11040441)
Supplement: Supplementary file 1 [file micromachines-11-00441-s001.zip › micromachines-732512- si/micromachines-732512-si proof.docx]

Supplementary Materials: Electrospun Fibers and Sorbents as a Possible Basis for Effective Composite Wound Dressings

Alan Saúl Alvarez-Suárez, Syed G. Dastager, Nina Bogdanchikova, Daniel Grande, Alexey Pestryakov, Juan Carlos Garcia Ramos, Graciela Lizeth Pérez-González, Karla Juárez-Moreno, Yanis Toledano-Magaña, Elena Smolentseva, Juan Antonio Paz-González, Tatiana Popova, Lyubov Rachkovskaya, Vadim Nimaev, Anastasia Kotlyarova^8^, Maksim Korolev, Andrey Letyagin^8^ and Luis Jesús Villarreal-Gómez


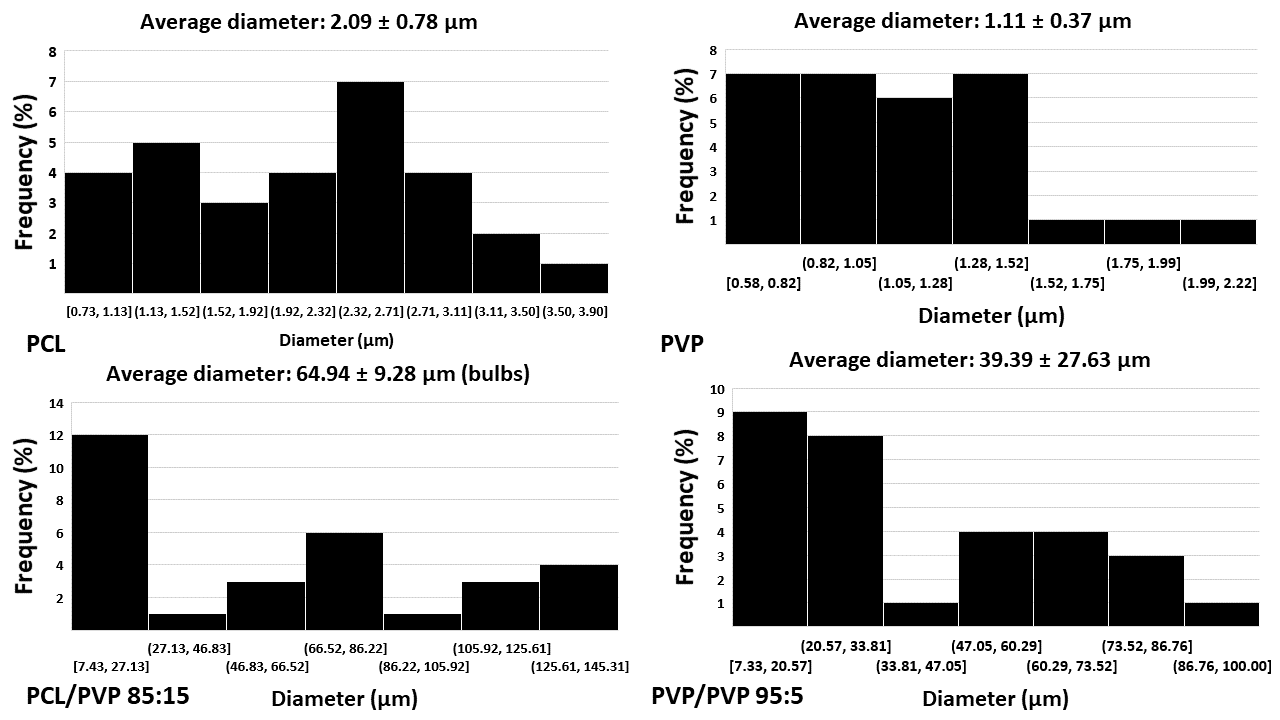


**Figure S1.** Average fiber diameter histograms for PCL, PVP, PCL/PVP 85:15 and PCL/PVP 95:5 microfibers.


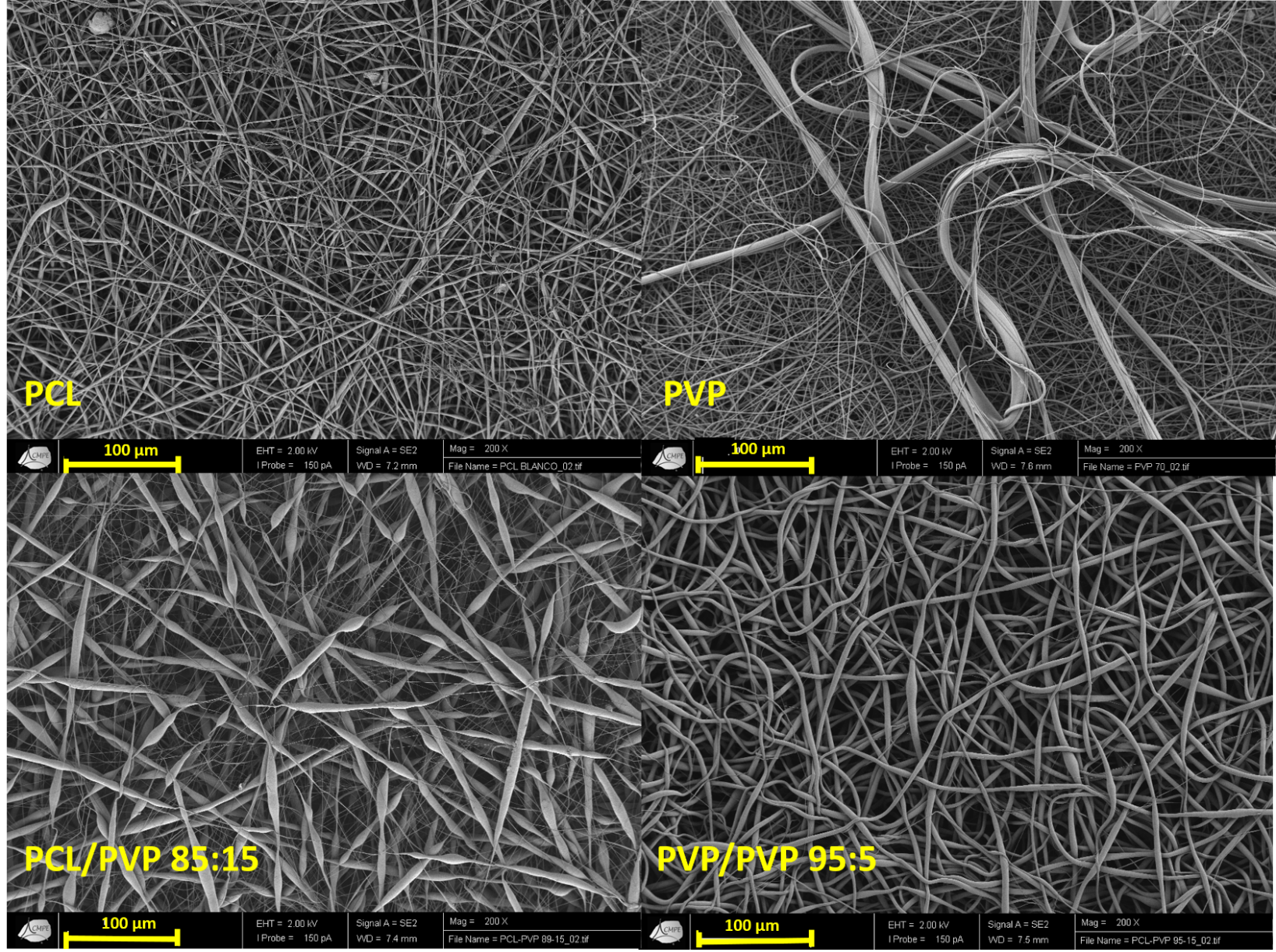


**Figure S2.** SEM images of PCL, PVP, PCL/PVP 85:15 and PCL/PVP 95:5 microfibers scaffolds. All pictures were acquired with an amplification of 200×, and were used to calculated average fiber diameter.


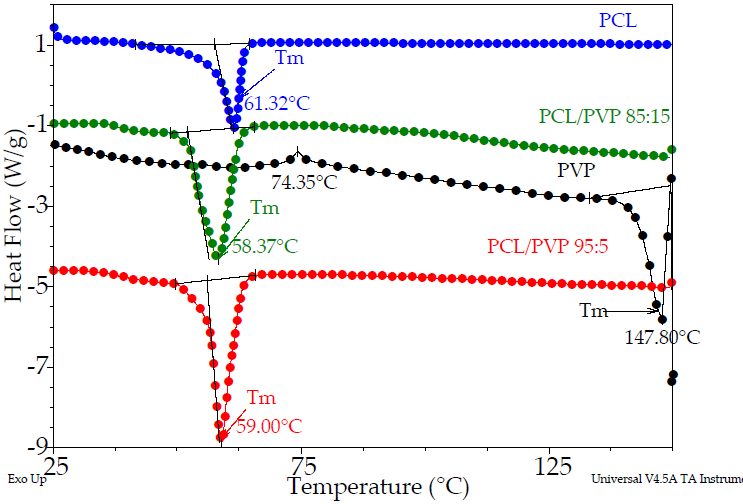


**Figure S3.** Differential Scanning Calorimetry thermograms of electrospun fibers produced with PCL, PVP, and PCL/PVP 95:5 and 85:15 ratio.


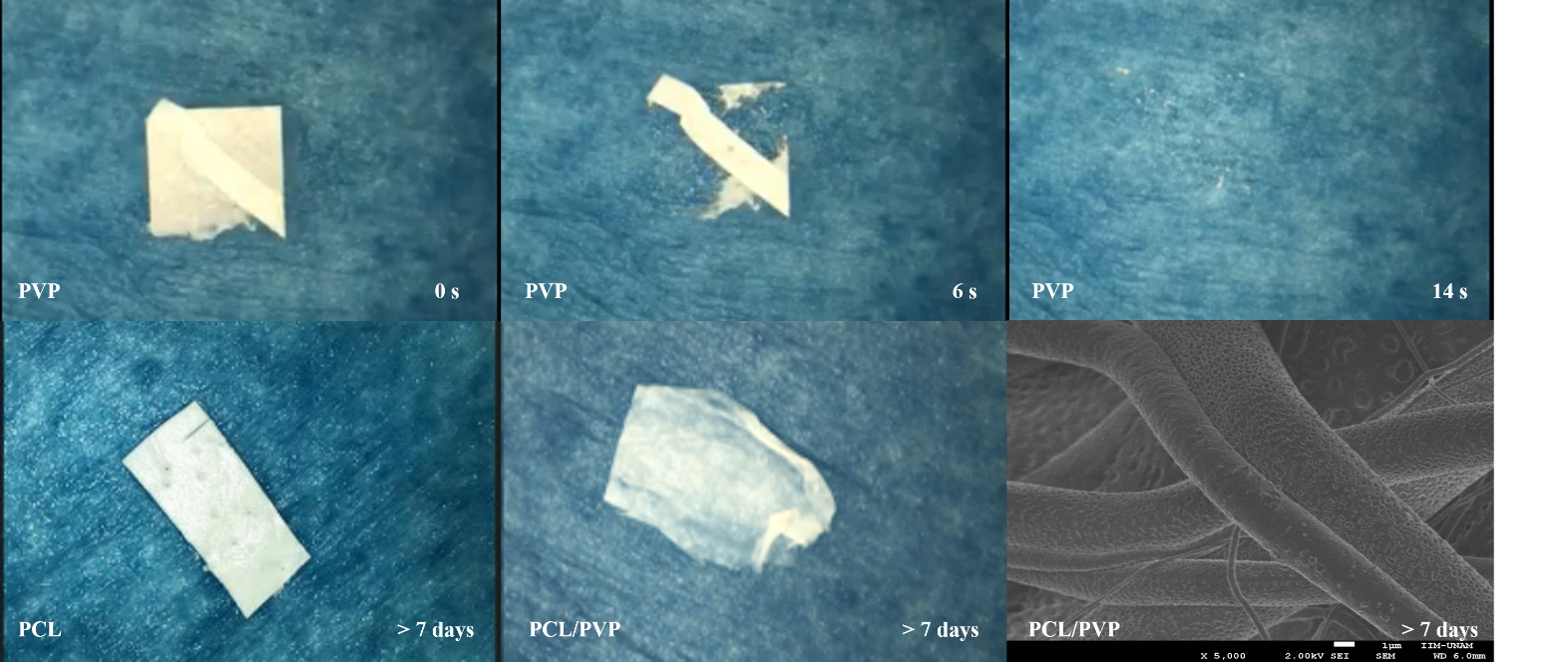


**Figure S4.** PCL, PVP, and PCL/PVP fibers dissolution test.


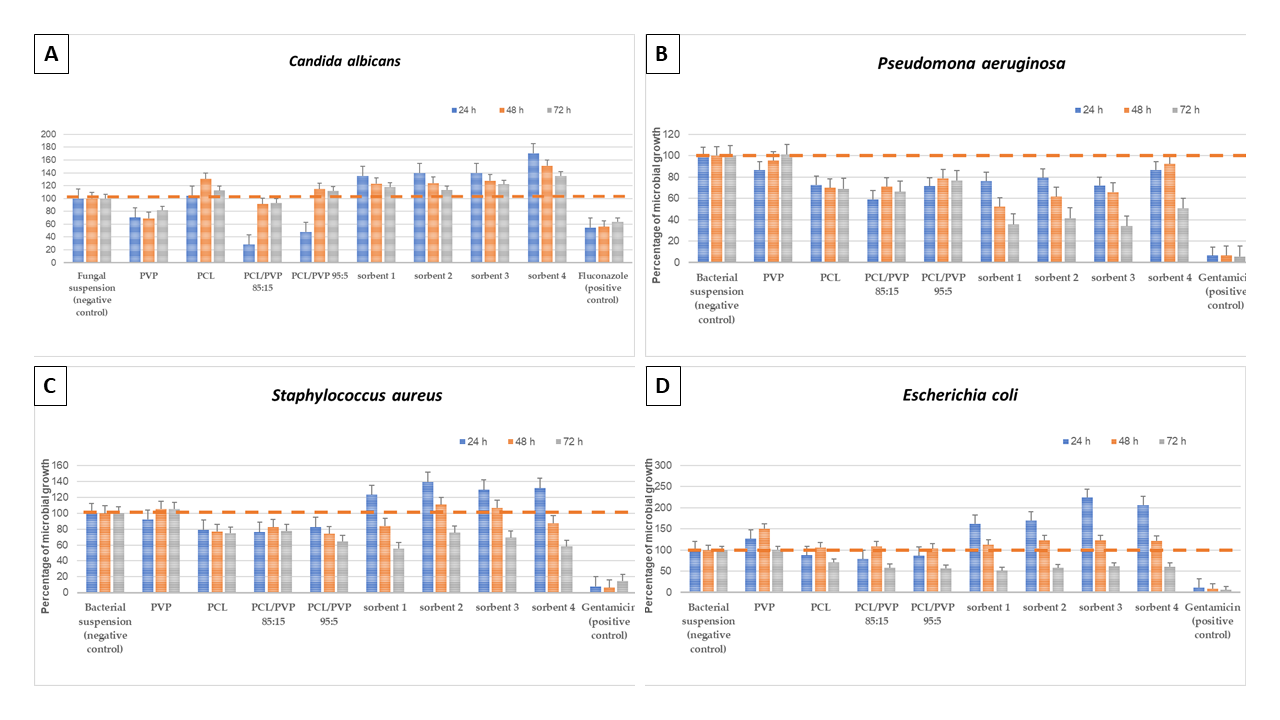


**Figure S5.** Percentage of microbial growth of exposed to (**A**) *Candida albicans;* (**B**) *Pseudomona aeruginosa*; (**C**) *Staphylococcus aureus*; (**D**) *Escherichia coli* to PCL/PVP fibers and Ag-Si/Al_2_O_3_ sorbents, at 24, 48 and 72 h.

**Table S1.** Percentage of microbial growth* at 24, 48 and 72 h after incubation with PCL/PVP microfibers and Ag-Si/Al_2_O_3_ sorbents. Measures were made by triplicate (average ± standard deviation).

|  | ***Pseudomona Aeruginosa***  **(% of growth)** | | | ***Staphylococcus Aureus***  **(% of growth)** | | | ***Escherichia***  ***Coli***  **(% of growth)** | | | ***Candida***  ***Albicans***  **(% of growth)** | | |
| --- | --- | --- | --- | --- | --- | --- | --- | --- | --- | --- | --- | --- |
|  | 24 h | 48 h | 72 h | 24 h | 48 h | 72 h | 24 h | 48 h | 72 h | 24 h | 48 h | 72 h |
| PVP | 87 ± 4 | 95 ± 2 | 101 ± 3 | 92 ± 4 | 105 ± 2 | 106 ± 8 | 127 ± 6 | 150 ± 3 | 100 ± 13 | 71 ± 5 | 69 ± 5 | 81 ± 5 |
| PCL | 73 ± 11 | 70 ± 11 | 69 ± 2 | 79 ± 3 | 77 ± 3 | 75 ± 1 | 88 ± 7 | 106 ± 4 | 71 ± 5 | 104 ± 14 | 130 ± 9 | 112 ± 13 |
| PCL/PVP 85:15 | 59 ± 9 | 71 ± 6 | 66 ± 3 | 77 ± 4 | 83 ± 4 | 78 ± 7 | 79 ± 4 | 108 ± 3 | 58 ± 3 | 28 ± 3 | 81 ± 9 | 93 ± 17 |
| PCL/PVP 95:5 | 71 ± 24 | 79 ± 9 | 76 ± 2 | 83 ± 7 | 74 ± 7 | 64 ± 5 | 87 ± 9 | 104 ± 5 | 57 ± 2 | 48 ± 4 | 114 ± 14 | 112 ± 24 |
| Ag-Si/Al_2_O_3_-1 | 76 ± 6 | 52 ± 13 | 36 ± 2 | 123 ± 10 | 84 ± 16 | 55 ± 19 | 162 ± 4 | 112 ± 5 | 51 ± 7 | 135 ± 18 | 123 ± 5 | 118 ± 2 |
| Ag-Si/Al_2_O_3_-2 | 79 ± 11 | 62 ± 7 | 42 ± 2 | 139 ± 17 | 111 ± 6 | 76 ± 3 | 170 ± 3 | 123 ± 3 | 58 ± 4 | 140 ± 13 | 124 ± 10 | 113 ± 3 |
| Ag-Si/Al_2_O_3_-3 | 72 ± 9 | 66 ± 9 | 34 ± 2 | 130 ± 3 | 107 ± 6 | 69 ± 4 | 224 ± 13 | 123 ± 1 | 62 ± 2 | 140 ± 45 | 128 ± 16 | 122 ± 9 |
| Ag-Si/Al_2_O_3_-4 | 87 ± 8 | 93 ± 2 | 51 ± 4 | 132 ± 11 | 88 ± 14 | 58 ± 15 | 206 ± 25 | 122 ± 4 | 61 ± 1 | 170 ± 23 | 151 ± 12 | 135 ± 2 |
| Gentamicin | 6 ± 0 | 7 ± 3 | 6 ± 0 | 8 ± 1 | 6 ± 1 | 15 ± 9 | 11 ± 0 | 9 ± 0 | 6 ± 0 | − | − | − |
| Fluconazole | − | − | − | − | − | − | − | − | − | 54 ± 2 | 56 ± 6 | 63 ± 9 |
| Microbial suspension | 100 | 100 | 100 | 100 | 100 | 100 | 100 | 100 | 100 | 100 | 100 | 100 |
| *All microbial growths were calculated comparing the obtained proliferation of the sample´s exposed cells with the normal growth if the microbial cell withour any alteration in the cell suspension that serves as a negative control. Each percentage value was calculated with respective control at each day of incubation. | | | | | | | | | | | | |
